# Neurosurgical Outcomes for Intracerebral Hemorrhage in Patients Undergoing Dialysis

**DOI:** 10.3390/life14111366

**Published:** 2024-10-24

**Authors:** Takuma Maeda, Mayuko Miyata, Nobuaki Naito, Koki Onodera, Yushiro Take, Aoto Shibata, Kaima Suzuki, Hidetoshi Ooigawa, Hiroki Kurita

**Affiliations:** Department of Cerebrovascular Surgery, Saitama Medical University International Medical Center, Hidaka 350-1298, Japan

**Keywords:** hemodialysis, intracerebral hemorrhage, neurosurgery, rebleeding, seizure, surgical evacuation

## Abstract

Patients on hemodialysis (HD) are at a very high risk of stroke, especially hemorrhagic stroke, with worse outcomes than the general population. We have determined the indications for urgent neurosurgery for intracerebral hemorrhage (ICH) based on the hematoma volume and neurological severity, regardless of HD status. This study aimed to evaluate the neurosurgical outcomes of ICH in patients undergoing HD. We retrospectively reviewed 38 cases of surgical removal of ICH performed in patients on HD. Patients were categorized into poor or better (0–4) and very poor (5 or 6) groups according to their modified Rankin Scale (mRS) score at discharge. Patient demographics, clinical characteristics, and operative records were retrospectively analyzed. The median Glasgow Coma Scale (GCS) score and hematoma volume were 6 and 99 mL, respectively. A total of 30 patients (78.9%) had very poor outcomes at discharge. Significant differences were observed in GCS score (13 vs. 6) and hematoma volume (53 vs. 114 mL) between the poor or better and very poor groups. The receiver operating characteristic curve analysis showed the cut-off values were 9 for GCS (AUC = 0.821) and 63.3 mL for hematoma volume (AUC = 0.812). The most common complication was rebleeding (10.5%), followed by seizures (7.9%), infection (7.9%), and cerebral edema (7.9%). In conclusion, neurosurgical outcomes of ICH in patients undergoing HD remain poor, but 21.1% of these patients achieved an mRS ≤ 4. ICH patients on HD with a GCS score > 9 or hematoma volume < 63 mL are more likely to demonstrate mRS ≤ 4 after surgical evacuation. The postoperative management of patients on HD should be performed considering specific risks, such as seizures and rebleeding.

## 1. Introduction

Hemodialysis (HD) is the most common form of kidney replacement therapy worldwide [1]. Access to HD has increased significantly, particularly in high-income countries [2]. Consequently, the number of patients with stroke receiving HD is also increasing [3]. Patients undergoing maintenance HD have a high incidence of intracerebral hemorrhage (ICH) and poor prognosis [4]. A previous study showed that the incidence of ICH in HD patients was higher than that of cerebral infarction (52% vs. 41%) [5]. In addition, the mortality rate from ICH is up to three times higher than from cerebral infarction, making ICH one of the leading causes of death in HD patients [6]. Several factors may contribute to the poor prognosis of patients with ICH undergoing HD, including active bleeding and hematoma expansion due to systemic anticoagulant therapy and severe cerebral edema, caused by the rapid osmolar shift due to the dialysis disequilibrium syndrome [7]. Surgical evacuation and postoperative management of ICH patients undergoing HD can be challenging due to these specific risk factors, and no guidelines have described on how to determine the indications for urgent neurosurgery in patients undergoing HD. In addition, very few reports have evaluated the neurosurgical outcomes for ICH in patients on HD [8,9].

In this study, we investigated the neurological outcomes of ICH in patients undergoing HD. We determined the indications for urgent neurosurgery for ICH based on the neurological severity and hematoma volume, regardless of HD status. We aimed to evaluate the neurosurgical outcomes and identify the risk factors for very poor outcomes in patients with ICH undergoing HD.

## 2. Materials and Methods

### 2.1. Patients and Study Design

We retrospectively reviewed the medical records of all patients (*n* = 38) with HD who underwent surgical removal of ICH at our institution between April 2007 and December 2023. The study protocol was reviewed and approved by Saitama Medical University International Medical Center (approval number, 2023-142). Written informed consent was waived because information from routine clinical practice was used. Patients with only intraventricular hemorrhage were excluded from the study. Patients were divided into two groups based on their clinical outcomes at discharge as follows: poor or better (0–4) and very poor (5 or 6), according to the modified Rankin Scale (mRS) [10]. These two groups were compared for the following factors: patient age, sex, medical history, oral antithrombotic medication, duration of HD, Glasgow Coma Scale (GCS) score on admission, hematoma volume, hematoma enlargement, location (putamen, thalamus, lobar region, and cerebellum), ventricular perforation, operative details (duration, blood transfusion, and complications including rebleeding), and length of hospital stay.

All ICH cases were detected using computed tomography (CT). Patients also underwent CT angiography, magnetic resonance imaging, or digital subtraction angiography to investigate the bleeding source. After these studies, neurologists and neurosurgeons discussed the indications for surgical removal according to the national stroke guidelines [11], regardless of HD status. In general, patients with small ICH (<10 mL) or ICH with minimal neurological deficit did not undergo surgery. At least one board-certified neurosurgeon was present during the surgical procedure. The hematoma was surgically removed under general anesthesia. Perioperative HD was performed according to the Japanese Society for Dialysis Therapy guidelines. HD was avoided within 24 h of ICH onset, except for severe hyperkalemia (serum K ≥ 6.0 mEq/L) or pulmonary edema. Perioperative HD was performed with reduced efficiency to minimize the effects of intracranial pressure. Hemofiltration (HF) was performed during the acute phase, and replacement fluid (Sublood BSG, Fuso, Tokyo, Japan) for HF was delivered at 20 L/session. Continuous HD was performed in patients with hemodynamic instability. Anticoagulation during dialysis was performed either with nafamostat mesylate or without anticoagulant.

### 2.2. Statistical Analysis

Quantitative variables are expressed as mean ± standard deviation. The x^2^ test or Fisher’s exact test was used to identify covariates that could be used as binary categorical dependent variables. Unpaired sample tests using Welch’s correction were used for parametric data, and Mann–Whitney U tests were used for nonparametric data. Statistical significance was set at *p* < 0.05. SPSS version 24 (IBM Corp., Armonk, New York, NY, USA) was used for all the statistical analyses.

## 3. Results

### 3.1. Baseline Characteristics

The baseline patient characteristics are summarized in Table 1. The median patient age was 63 (interquartile range [IQR], 56–71) years and more males (71.1%) than females (28.9%) were included. Most patients (89.5%) had hypertension, and nine (23.7%) patients had a previous stroke. Thirteen (34.2%) patients were taking oral antithrombotic medications. The median duration of dialysis was 9 (IQR, 4–15) years. Diabetic nephropathy (39.5%) was the most common HD cause, followed by chronic glomerulonephritis (18.4%), immunoglobulin A nephropathy (7.9%), polycystic kidney disease (5.3%), chronic interstitial nephritis (2.6%), and nephrosclerosis (2.6%). No significant differences in patient characteristics between the two groups were observed.

### 3.2. Clinical Features and Outcomes

**Figure 1:**
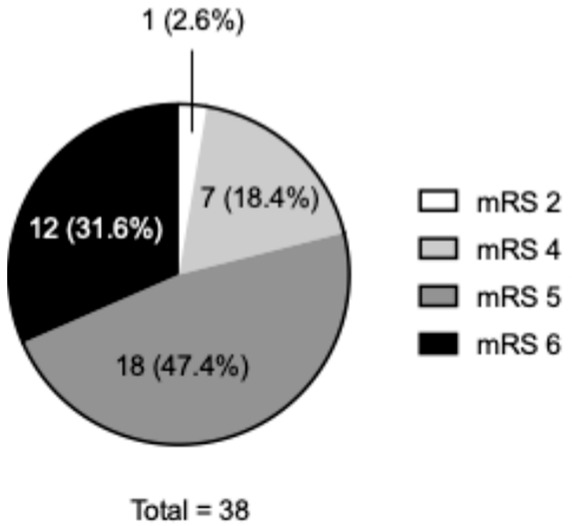
Modified Rankin Scale (mRS) score at discharge.

Clinical features and outcomes are summarized in Table 2 and Figure 1. The median GCS score was 6 (IQR, 4–13). The median hematoma volume was 99 (IQR, 55–167) mL, with 15.8% of the hematomas increasing in size prior to surgical removal. The most common location of the hematoma was the putamen (57.9%), followed by the lobar region (31.6%). Ventricular perforation was observed in 76.3% of the patients. The median intraoperative blood loss was 320 (IQR, 164–444) mL and 44.7% of the patients required intraoperative blood transfusion. Approximately half of the patients experienced perioperative complications. The most complications were rebleeding (10.5%), followed by seizures (7.9%), infection (7.9%), and severe cerebral edema (7.9%). A total of 30 patients (78.9%) had a very poor outcome, including 12 patients (31.6%) who died in hospital.

### 3.3. Predictors of Clinical Outcomes

**Figure 2:**
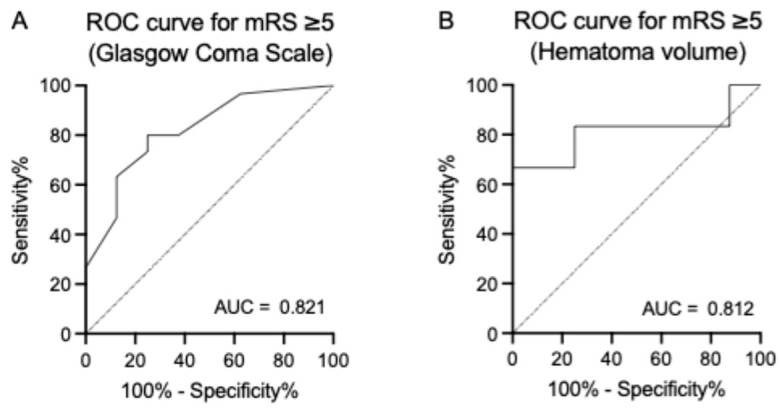
Receiver operating characteristic (ROC) curve for predicting very poor outcomes based on the Glasgow Coma Scale (GCS, (**A**)) and hematoma volume (**B**). The area under the curve was 0.821 and 0.812 for GCS and hematoma volume, respectively.

Significant differences were observed in the GCS score (13 vs. 6; *p* < 0.01) and hematoma volume (53 vs. 114 mL; *p* < 0.01) between the poor or better and very poor groups. Receiver operating characteristic (ROC) curve analysis showed that the cutoff values for very poor outcomes were 9 for GCS (area under the curve [AUC] = 0.821) and 63.3 mL for hematoma volume (AUC = 0.812) (shown in Figure 2). Regarding the location of the hemorrhage, putaminal hemorrhage was a predictor of very poor outcomes (25.0% vs. 66.7%; *p* < 0.05), while lobar hemorrhage was a predictor of poor or better outcomes (75.0% vs. 20.0%; *p* < 0.01). Operative time was significantly longer in the very poor group (145 vs. 214 min; *p* < 0.05). No rebleeding occurred within 30 days after the procedure in the poor or better group. Although no significant differences in intraoperative blood loss were observed (245 vs. 321 mL; *p* = 0.485), intraoperative blood transfusions tended to be more frequent in the very poor group (25.0% vs. 50.0%; *p* = 0.258). Perioperative complications (25.0% vs. 53.3%; *p* = 0.238) and length of hospital stay (24 vs. 35 days; *p* = 0.531) were higher in the very poor group. However, these differences were not statistically significant.

## 4. Discussion

Herein, we described the neurosurgical outcomes for ICH in patients undergoing HD in the current settings. We showed the cutoffs of ICH volume and GCS for very poor outcomes after surgery. Furthermore, we demonstrated the correlation between the location of the hematoma and the subsequent prognosis. A previous study demonstrated that FUNC score and intraventricular extension at admission were predictors of a very poor outcome (mRS ≥ 5) in all ICH patients on HD [12]. Although the FUNC score was not investigated in our study, the predictors identified (ICH volume, GCS, and hematoma location) constitute the primary components of the FUNC score. Conversely, the incidence of intraventricular hemorrhage resulting in intraventricular extension was not identified as a predictor of outcome in the present study. This may be due to the fact that intraventricular extension can be effectively addressed through intraoperative irrigation or drainage of the ventricle, in surgical cases.

Approximately 4 million people worldwide receive kidney replacement therapy, and HD remains the most common form. In Japan, the number of patients receiving dialysis was 349,700 by the end of 2021, with this number increasing annually [13]. Approximately 1 in 360 individuals in Japan are dependent on dialysis, making Japan the second most prevalent country in the world. Neurosurgical emergencies in patients with stroke undergoing HD are not rare. Renal dysfunction is a major risk factor for stroke and contributes significantly to stroke morbidity and mortality [3,14]. Specifically, the incidence of stroke is 8–10 times higher in patients on dialysis, and mortality has also increased [15,16]. The incidence of stroke in patients on dialysis is reported to be 10–35 per 1000 person-years, with hemorrhagic stroke accounting for 20–30% of all strokes [3,15,16]. Patients with chronic renal failure stage 3–5D, including patients on dialysis, have poor outcomes and decreased survival rates after stroke [17,18,19]. The increased risk of stroke in patients on dialysis is reported to be due to the interaction between vascular complications caused by renal dysfunction and pathological conditions specific to uremia, such as vascular calcification and the malnutrition-inflammation-atherosclerosis syndrome [3,5]. Oral anticoagulants and antiplatelet agents are cornerstones of stroke prevention. However, the hemorrhagic tendency is more pronounced in patients undergoing HD; hence, a balance between stroke prevention and bleeding risk must be considered. In addition, these patients are prone to hematoma enlargement due to anticoagulant therapy, and urgent neurosurgery is often undertaken to remove the hematoma. No guidelines or consensus exist on how to determine the indications for urgent neurosurgery in patients on dialysis with such a background because their natural history is unknown. According to the dialysis therapy guidelines for the management of cardiovascular disease, neurosurgery for ICH has a weak recommendation and low levels of evidence [20]. In Japan, the National Cerebral and Cardiovascular Center reported in the 1990s that the postoperative mortality rate of urgent neurosurgery for ICH in patients on HD was 60% and the rebleeding rate was 40%, although the sample size was small [8]. Thus, higher mortality and rebleeding rates in patients undergoing dialysis have been previously reported. In this study, the mortality rate was 31.6% and the rebleeding rate was 10.5%, indicating a decrease in mortality and rebleeding rates compared to previous studies. This may be largely due to advances in dialysis technology, anticoagulants, and their antagonists [21,22,23].

The proportion of ICH patients with very poor outcomes (mRS score: 5–6) at discharge tended to be higher in patients on dialysis (78.9% in our study) than in the general population (35.8%), as reported by the Japan Stroke Data Bank 2023 “https://strokedatabank.ncvc.go.jp/en/ (accessed on 20 October 2024)”. According to the Diagnosis Procedure Combination in Japan, the mortality rate after the surgical procedure for ICH was 7.5–17.4% in the general population [24]. Although these results are not directly comparable with those of our study, the neurosurgical outcomes of patients undergoing HD seem to be worse than those of the general population. We investigated the predictors of very poor outcomes in this context. The GCS score and hematoma volume were predictors of clinical outcomes after surgical removal of ICH. ROC analysis revealed that a GCS score of 9 and hematoma volume of 63 mL were cutoffs for predicting very poor outcomes after surgery.

Postoperative seizures and rebleeding should be considered specific complications of perioperative management. In the present study, rebleeding was the most common perioperative complication. In patients on dialysis, anticoagulant and antiplatelet drugs are often introduced due to risk factors for cardiovascular diseases, such as hypertension, hyperlipidemia, and diabetes mellitus, suggesting their involvement in the formation and growth of ICH and postoperative rebleeding [25]. β2-microglobulin-induced renal dialysis-induced amyloidosis may also contribute to the cerebrovascular vulnerability in patients on HD [26]. Maintenance dialysis has also been reported as a risk factor for postoperative seizures during neurosurgery [27]. In our study, acute seizures were the second most common perioperative complication. Although the reason for this has not been identified, this may be due to the fact that electrolyte imbalance and hypotension easily occur in patients undergoing dialysis because of the rapid clearance from the blood compared to the cerebrospinal fluid, uremia, and metabolic disorders associated with end-stage renal failure, as well as additional surgical invasiveness [28]. Therefore, the prophylactic administration of anticonvulsants before and after urgent neurosurgery in patients on HD may be useful for improving outcomes. However, the timing and blood concentration of anticonvulsants, such as levetiracetam and lacosamide, which have been widely used in recent years, should be carefully monitored because of their high clearance by dialysis [29].

### Limitations

This study has several limitations. First, this was a single-center retrospective non-randomized study with a limited number of patients. Second, direct comparison between the non-dialysis and non-surgical patients was lacking, making statistical investigation difficult. Third, neurosurgeons were not standardized, and outcomes may have been influenced by their individual learning curves, although at least one board-certified neurosurgeon was present throughout the surgical procedure to ensure the quality of the surgery. Therefore, concluding the efficacy and safety of the surgical removal of ICH in patients on HD, based solely on the results of this study, is challenging. Nevertheless, we believe that this study will help neurosurgeons consider the indications for surgical removal of ICH in patients undergoing HD. Further studies are needed, including prospective studies and comparisons with non-dialysis patients.

## 5. Conclusions

Although the neurological outcomes of ICH in patients on HD remain poor, 21.1% of these patients achieved an mRS ≤ 4 after surgical evacuation. Based on our results, ICH patients on HD with a GCS score > 9 or hematoma volume < 63 mL are more likely to demonstrate mRS ≤ 4 after surgical evacuation. Postoperative management of HD patients should consider specific risks, such as seizures and rebleeding.

## Figures and Tables

**Table 1:** Baseline characteristics of all 38 patients with intracerebral hemorrhage on dialysis.

	All	Poor or Better	Very Poor	*p* Value
No. of patients	38 (100)	8 (21.1)	30 (78.9)	
Age (yrs), median [IQR]	63 [56–71]	60 [53–64]	64 [56–71]	0.390
Female sex	11 (28.9)	3 (37.5)	8 (26.7)	0.667
Past medical history				
Hypertension	34 (89.5)	8 (100)	26 (86.7)	0.560
Diabetes	15 (39.5)	3 (37.5)	12 (40.0)	1.000
Hyperlipidemia	3 (7.9)	1 (12.5)	2 (6.7)	0.519
Any stroke	9 (23.7)	1 (12.5)	8 (26.7)	0.650
Oral antithrombotic medication	13 (34.2)	1 (12.5)	12 (40.0)	0.222
Antiplatelets	11 (28.9)	1 (12.5)	10 (33.3)	0.395
Anticoagulants	3 (7.9)	1 (12.5)	2 (6.7)	0.519
Basic disease for dialysis				
Chronic glomerulonephritis	7 (18.4)	0 (0)	7 (23.3)	0.307
Chronic interstitial nephritis	1 (2.6)	1 (12.5)	0 (0)	0.211
Diabetic nephropathy	15 (39.5)	3 (37.5)	12 (40.0)	1.000
IgA nephropathy	3 (7.9)	1 (12.5)	2 (6.7)	0.519
Nephrosclerosis	1 (2.6)	0 (0)	1 (3.3)	1.000
Polycystic kidney disease	2 (5.3)	0 (0)	2 (6.7)	1.000
Others	9 (23.7)	3 (37.5)	6 (20.0)	0.363
Duration of dialysis (yrs), median [IQR]	9 [4–15]	8 [6–16]	9.0 [4–14]	1.000

Values are number (%) except where indicated otherwise. IQR, interquartile range.

**Table 2:** Clinical features and outcomes of patients with intracerebral hemorrhage on dialysis.

	All	Poor or Better	Very Poor	*p* Value
Glasgow Coma Scale, median [IQR]	6 [4–13]	13 [10–14]	6 [3–8]	0.006
Hematoma volume (mL), median [IQR]	99 [55–167]	53 [50–65]	114 [79–170]	0.008
Hematoma enlargement	6 (15.8)	4 (50.0)	2 (6.7)	0.012
Location				
Putamen	22 (57.9)	2 (25.0)	20 (66.7)	0.049
Thalamus	3 (7.9)	0 (0)	3 (10.0)	1.000
Lobar	12 (31.6)	6 (75.0)	6 (20.0)	0.007
Cerebellum	2 (5.3)	0 (0)	2 (6.7)	1.000
Venticular perforation	29 (76.3)	5 (62.5)	24 (80.0)	0.363
Operative time (min), median [IQR]	200 [163–259]	145 [98–189]	214 [176–269]	0.012
Intraoperative blood loss (mL), median [IQR]	320 [164–444]	245 [98–420]	321 [166–442]	0.485
Intraoperative blood transfusion	17 (44.7)	2 (25.0)	15 (50.0)	0.258
Perioperative complication	18 (47.4)	2 (25.0)	16 (53.3)	0.238
Rebleeding *	4 (10.5)	0 (0)	4 (13.3)	0.560
Seizure	3 (7.9)	1 (12.5)	2 (6.7)	0.519
Infection	3 (7.9)	1 (12.5)	2 (6.7)	0.519
Severe cerebral edema	3 (7.9)	0 (0)	3 (10.0)	1.000
Hospital stay (day), median [IQR]	28 [12–48]	24 [23–28]	35 [9–65]	0.531

* Rebleeding within 30 days after the procedure. Values are number (%) except where indicated otherwise. IQR, interquartile range.

## Data Availability

The data presented in this study are available on request from the corresponding author.
